# Genotoxicity of physical silver nanoparticles, produced by the HVAD method, for *Chinchilla lanigera* genome

**DOI:** 10.1038/s41598-021-97926-9

**Published:** 2021-09-16

**Authors:** Anna Grzesiakowska, Marek Jan Kasprowicz, Marta Kuchta-Gładysz, Katarzyna Rymuza, Olga Szeleszczuk

**Affiliations:** 1 Department of Animals Reproduction, Anatomy and Genomics, University of Agriculture in Krakow, Mickiewicza Av. 24/28, 30-059 Kraków, Poland; 2 Department of Soil Science and Agrophysics, University of Agriculture in Krakow, Mickiewicza Av. 21, 31-120 Kraków, Poland; 3 Faculty of Agrobioengineering and Animal Husbandry, Siedlce University of Natural Sciences and Humanities, ul. B. Prusa 14, 08-110 Siedlce, Poland

**Keywords:** Genetics, Environmental sciences, Nanoscience and technology

## Abstract

Each year, growing demand for silver nanoparticles (AgNP) contributes to the search for alternative methods of their production. Stable AgNP with antibacterial properties, low toxicity to the environment and living organisms are especially valued. In the study presented here, an attempt was made to assess the toxicity of two AgNP solutions produced using the HVAD method to the *Chinchilla lanigera* genome. The AgNO_3_ solution was the indicator and reference for the harmfulness of AgNP. The study was carried out in vitro on bone marrow cells isolated from *Chinchilla lanigera* bones. The genotoxicity was assessed by comet assay, following the treatment of cells with three silver solutions: unstable and sodium citrate-stabilized silver nanoparticles, as well as silver nitrate at three concentrations (5, 10 and 20 µg/L), after 3, 6 and 24 h. Based on the percentage of the DNA content in the comet tail and the tail moment, an increase in cell DNA integrity disruption was demonstrated in all tested variants: of solution, exposure time and concentration, compared to the control sample. A statistically significant correlation was determined between the level of induced DNA breaks and the concentration of the active solutions and the duration of their activity. A solution of silver nanoparticles stabilized with sodium citrate was shown to have the most harmful effect on bone marrow cells. Silver nitrate demonstrated a level of toxicity similar to these particles. Further studies are necessary to directly compare the genotoxic properties of AgNP produced using the HVAD method and the chemical method under the same conditions.

## Introduction

The antibacterial properties of silver nanoparticles (AgNP) increase the interest in products containing this compound. AgNP is increasingly frequently used in various industries, such as cosmetic, textile, food and construction^1,2^. Silver nanoparticles are also used in human and veterinary medicine, e.g. for the production of wound dressings, as a drug carrier, in microsurgery and in surgical instruments^3–5^.

The small size of its nanoparticles (one of the sizes below 100 nm) enables AgNP to access the body by inhalation or skin penetration, then to enter the cell^1,6^. There, silver nanoparticles accumulate, blocking the cell cycle, inducing oxidative stress and leading to cell apoptosis. Accumulated in cells, they can disturb the proper functioning of the body, thus indicating AgNP toxicity. An important aspect in the research on metal nanoparticles, including silver, resulting from the properties of the particles and their interaction with cells or living organisms, is their potential genotoxicity or cytotoxicity. They are associated with the possibility of DNA damage, chromosomal changes and induction of mutagenic processes^4,7^.

One of the factors influencing the potential toxicity and the properties of nanoparticles is the method in which they are obtained^8^. Silver nanoparticles can be generated biologically. The ability to synthesize AgNP is demonstrated by bacteria, fungi and plants^9^. Most of the industrially used AgNP is obtained by chemical methods. The techniques most commonly used for this purpose involve chemical reduction in aqueous or alcoholic solutions, enabling obtaining nanoparticles at a low cost. The disadvantage of the chemical methods is the use of harmful chemicals, which may adversely affect the environment^4^. Indirect methods of obtaining silver nanoparticles are also available, such as the electrochemical method^8^. As a result of the search for techniques enabling the obtaining of more uniform nanoparticles without residual chemical compounds, physical methods of AgNP synthesis are being developed^8,9^. One of the physical techniques is the High Voltage Arc Discharge (HVAD) method^10^. It enables obtaining small sized AgNP, which in contrast to those derived from chemical reduction, are not contaminated with chemical compounds.

Cytogenetic tests such as the comet assay or the micronucleus assay are used to assess the genotoxicity of substances including silver nanoparticles^11–15^. These are tools enabling quick and effective assessment of the effects of AgNP on cells and living organisms.

The comet assay, also known as single cell gel electrophoresis (SCGE), is a simple, quick and sensitive method of detecting and assessing the degree of DNA fragmentation resulting from genotoxic factors. By this method, the degree of DNA damage and repair may be analyzed at the level of individual cells of a given organism and species^5,16^. Fragments of damaged DNA are migrated from the nucleus during the electrophoretic division, which may be assessed visually in microscopic examination^14^. The alkaline comet assay enables the analysis of both single strand breaks (SSB) and double strand breaks (DSB), alkali-labile sites (ALS), and other chemical and enzymatic modifications, which can transform into DNA breaks^14,17^.

The purpose of the study was to check whether silver nanoparticles produced using the physical method (HVAD) show genotoxic effect on bone marrow cells. It has been hypothesized that the concentration of the tested solutions and the time of exposure significantly affect the level of cell DNA integrity disruption.

## Results

**Figure 1:**
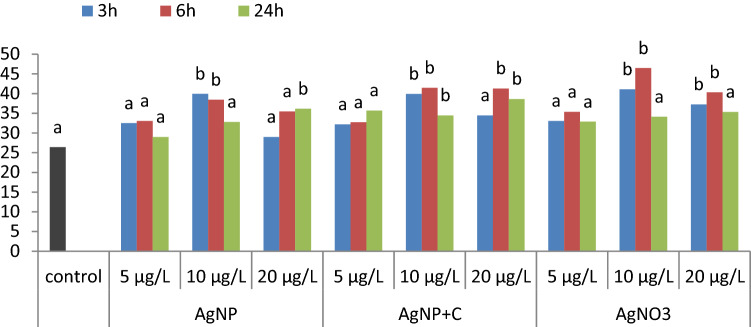
The mean percent content of DNA in the comet tail for the control samples and the cells tested at various concentrations, after 3, 6 and 24 h.

As a result of the comet assay in the control, cell damage was determined at the level of 26.43 ± 10.29% of the DNA in the comet's tail (Fig. 1).

The average number of helix breaks (% DNA Tail) in the DNA treated with the analyzed solutions (AgNP, AgNP + C and AgNO_3_) at concentrations of 5 µg/ L for 3, 6 and 24 h did not differ significantly from the average cell damage in the control sample (Fig. 1).

**Figure 2:**
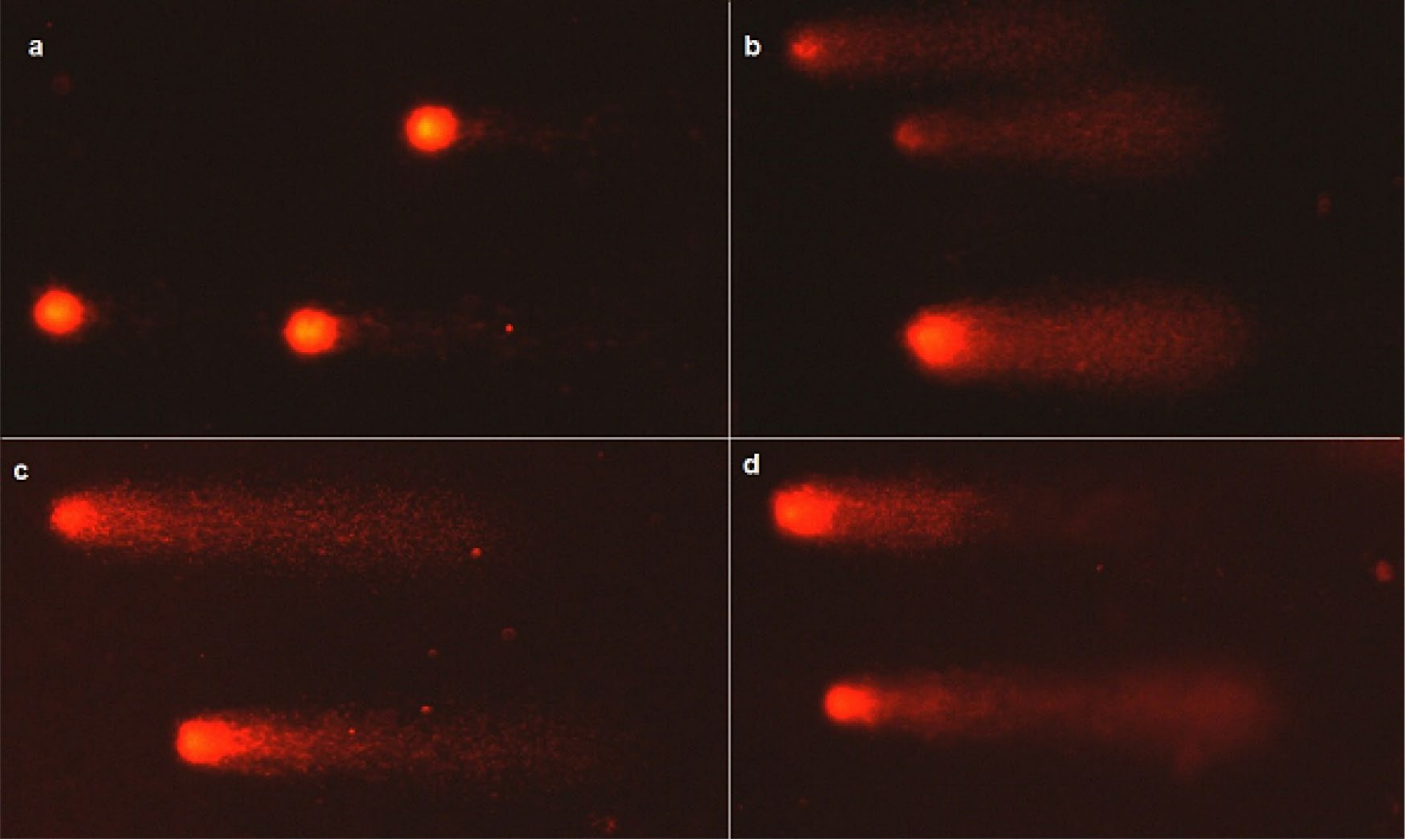
Bone marrow cells of *Chinchilla lanigera* with different levels of DNA damage in the comet assay: (**a**) cells from control sample; (**b**) cells after exposure 6 h for AgNP 10 µg/L; (**c**) cells after exposure 6 h for AgNP + C 10 µg/L; (**d**) cells after exposure 6 h for AgNO_3_ 10 µg/L. 400 × magnification.

AgNP and AgNO_3_ solutions at the concentration of 10 µg/L caused significantly greater damage to cells after 3 and 6 h of operation when compared to controls. AgNP + C solution at the concentration of 10 µg/L had a negative impact on cells both after 3, 6 and 24 h of exposure (Figs. 1, 2).

As compared to the control sample, at the concentration of 20 µg/L, AgNP solution significantly increased the degree of cell DNA fragmentation after 24 h of exposure, AgNP + C solution did so after 6 and 24 h, while AgNO_3_ solution did so after 3 and 6 h.

The mean values marked with the letter a do not differ significantly from the control sample. The mean values marked with the letter b differ significantly from the control sample at *p* ≤ 0.05.

Changes in nuclear chromatin integrity, assessed on the basis of the percentage of DNA in the comet tail depending on the solutions used, their concentrations and exposure time are presented in Table 1.

**Table 1:** Genotoxicity of silver solutions based on the % DNA Tail value depending on the solutions, their concentrations and exposure time.

Solution	Time (h)	Concentration (µg/L)			Mean values for the solution
		5	10	20	
AgNP	3	32.51 ± 9.62	39.96 ± 11.97	29.00 ± 5.29	33.82 ± 10.22
	6	33.09 ± 6.58	38.45 ± 12.63	35.46 ± 11.48	35.67 ± 10.49
	24	29.02 ± 10.39	32.81 ± 12.19	36.20 ± 10.63	32.68 ± 11.18
	Mean	31.54 ± 8.94	37.07 ± 12.31	33.56 ± 9.83	34.06^b^ ± 10.62
AgNP + C	3	32.21 ± 6.15	39.93 ± 9.99	34.46 ± 9.26	35.53 ± 9.00
	6	32.76 ± 9.64	47.15 ± 10.71	41.30 ± 16.42	40.41 ± 13.63
	24	35.70 ± 11.69	41.49 ± 10.24	38.66 ± 8.19	38.62 ± 10.14
	Mean	33.56 ± 9.29	42.86 ± 10.50	38.14 ± 11.87	38.18^a^ ± 11.18
AgNO_3_	3	33.08 ± 8.76	41.11 ± 8.75	37.27 ± 5.66	37.15 ± 8.32
	6	35.39 ± 11.65	46.49 ± 8.35	40.33 ± 10.94	40.74 ± 11.11
	24	32.89 ± 11.86	34.17 ± 14.44	35.36 ± 12.73	34.14 ± 12.72
	Mean	33.79 ± 10.59	40.59 ± 11.73	37.65 ± 10.14	37.34^a^ ± 11.10
Average for concentration		32.96^C^ ± 9.60	40.17^A^ ± 11.68	36.45^B^ ± 10.75	36.52 ± 11.07
Average for exposure time		3 h	6 h	24 h	
		35.50^B^ ± 9.23	38.93^A^ ± 11.94	35.14^B^ ± 11.57	

a, b, c—values for solutions marked with different letters vary significantly (*p* ≤ 0.05).

A, B, C—Mean values for concentration and time marked with different letters differ significantly (*p* ≤ 0.5).

Statistical analysis showed that the value of the percentage of DNA Tail depends on the type of solution, concentration and exposure time. However, the interaction of the analyzed factors was not proven (Table 1).

During treatment with AgNP + C the most DNA helix breaks were formed, on average of 38.18% of DNA Tail. A comparable damage was obtained in cells treated with AgNP (34.06) and AgNO_3_ (37.34). While analyzing the effects of the concentration on the stability of bone marrow cell DNA, it was found that the greatest damage was caused by solutions at a concentration of 10 µg/L (40.17), less damage by solutions at a concentration of 20 µg/L (36.42), and the least damage by those at a concentration of 5 µg/L (32.96). Lack of relationship between the type of solution and the concentration indicates that the response of cells to increasing concentrations of the solutions was similar. Both for AgNP, AgNP + C and AgNO_3_, an increase in cell DNA damage was observed with the change in concentration from 5 to 10 µg/L, and then a decrease at a concentration of 20 µg/L. However, the insignificance of the interaction between the solution and the exposure time proves that cells in all solutions underwent similar damage due to the extension of the exposure time. The mean cell damage increased after 6 h of exposure and then decreased after 24 h.

**Figure 3:**
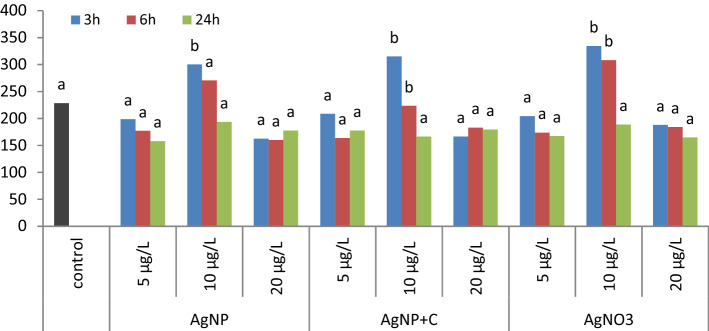
Mean cell damage level as TM values for the controls and tested samples at different concentrations after 3, 6 and 24 h.

The second of the analyzed parameters, indicating the genotoxic nature of the studied factors in the comet assay, was the tail moment (TM). The comet assay for the tail moment in the control sample demonstrated cell damage at the level of 84.01 ± 60.39 (Fig. 3). Damage similar to the control sample after 3, 6 and 24 h was observed following the use of tested solutions at the concentrations 5 and 20 µg/L. Mean values of cell damage at the tail moment were greater for cells treated with AgNP solution at a concentration of 10 µg/L for 3 h, with AgNP + C solution and AgNO_3_ at a concentration of 10 µg/L for the periods of 3 and 6 h (Fig. 3).

The mean values marked with the letter *a* do not differ significantly from the control sample. The mean values marked with the letter *b* differ significantly from the control sample at *p* ≤ 0.05.

The tail moment values significantly depended on the exposure time and concentration. However, no differences between the solutions were proven. The value of the TM parameter for the solutions ranged from 83.42 for AgNP to 94.81 for AgNP + C.

Comparing the effects of the concentration of the tested samples, regardless of the solution, no statistically significant differences were found between the concentrations of 5 µg/L (71.39 ± 31.87) and 20 µg/L (74.01 ± 31.23). The highest values of TM were observed for the concentration of 10 µg/L at the level of 127.03 ± 63.27. While analyzing the effect of the exposure time of bone marrow cells to the activity of silver solutions, it was found that extending the exposure time to 24 h resulted in a significant decrease in the level of genomic instability to the average of 77.29 ± 38.82 (Table 2).

**Table 2:** The genotoxicity of silver solutions based on the values of the Tail Moment, TM.

Solution	Time (h)	Concentration (µg/L)			Mean values for the solutions
		5	10	20	
AgNP	3	75.94 ± 27.52	145.13 ± 73.21	56.17 ± 17.64	92.41 ± 59.30
	6	66.78 ± 22.57	125.27 ± 64.86	69.80 ± 31.94	87.28 ± 50.46
	24	57.07 ± 27.89	78.46 ± 42.28	76.13 ± 34.44	70.55 ± 35.69
	Mean	66.59 ± 26.52	116.28 ± 66.12	67.37 ± 29.37	83.42 ± 49.88
AgNP + C	3	78.86 ± 20.92	148.84 ± 66.93	65.42 ± 25.13	97.71 ± 55.86
	6	63.86 ± 37.17	141.93 ± 55.61	89.38 ± 48.83	98.39 ± 56.94
	24	79.51 ± 40.82	106.06 ± 42.10	79.42 ± 25.06	88.33 ± 37.94
	Mean	74.07 ± 33.90	132.27 ± 57.44	78.07 ± 35.28	94.81 ± 50.73
AgNO_3_	3	78.13 ± 25.21	157.65 ± 55.93	75.70 ± 15.82	103.83 ± 52.46
	6	75.00 ± 41.03	160.09 ± 61.74	82.34 ± 29.66	105.81 ± 59.40
	24	67.38 ± 38.51	79.869 ± 49.78	71.75 ± 37.02	73.00 ± 41.27
	Mean	73.50 ± 34.87	132.54 ± 66.24	76.60 ± 28.38	94.21 ± 53.28
Average for concentration		71.39^B^ ± 31.87	127.03^A^ ± 63.27	74.01^B^ ± 31.23	90.81 ± 51.42
Average for exposure time		3 h	6 h	24 h	
		97.98^A^ ± 55.61	97.16^A^ ± 55.73	77.29^B^ ± 38.82	

A,B—mean values for concentration and exposure time marked with different letters vary significantly (*p* ≤ 0.05).

**Figure 4:**
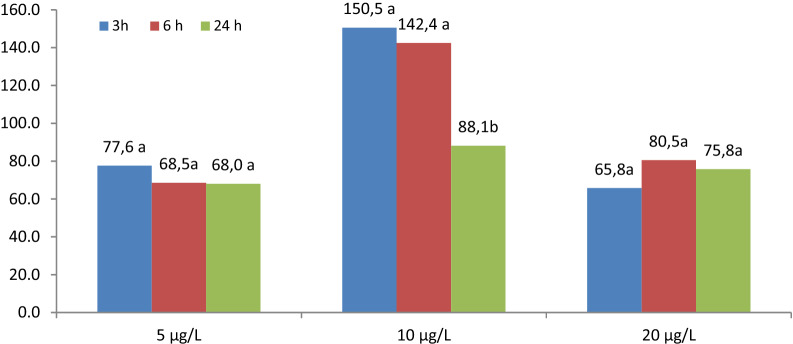
Value of the TM parameter depending on the concentration and exposure time.

Moreover, statistical analysis showed a significant interaction of concentration and exposure time, which indicates that the value of the TM parameter was different for the analyzed concentrations under the influence of exposure time (Fig. 4).

At the concentration of 5 µg/L, the values of the TM parameter were found to be at a comparable level along with the extension of the exposure time. At the concentration of 20 µg/L, exposure time was also irrelevant, as the differences between the mean damage values were not statistically significant (although their value ranged from 65.8 to 80.5). At the concentration 10 µg/L, extension of the exposure time to 24 h resulted in a significant reduction of the TM parameter to the level of 88.1. At this concentration, extending the exposure time from 3 to 6 h did not significantly reduce the value of TM.

## Discussion

Silver nanoparticles differ in the production method, particle size, concentration, shape or the form of coating. These features define the physicochemical properties of nanoparticles, which in turn may affect their toxicity to living organisms^12,18^. Due to the prevalence of AgNP in everyday products, their increased release and then bioaccumulation in the environment are a frequent occurrence. This may result in the penetration of AgNP into the food chain^1,11^. So far, there has been no information on the harmful effects of nanoparticles derived from household or industrial waste on the biological processes in human and animal organisms or plants exposed to them^4,19,20^. Due to the wide use of silver nanoparticles, there are considerable concerns about their continuous release into the environment of humans and animals^1,4,21,22^. At present, well-characterized, less toxic AgNPs are sought, produced by techniques other than common chemical methods. Despite the development of biological and physical methods of AgNP synthesis, there is little information about nanoparticles demonstrating favorable properties, and above all, lower toxicity. In the research conducted, a preliminary assessment of the toxicity of silver nanoparticles produced using the physical method, HVAD, was undertaken. This method makes use of the phenomenon of electrical discharges between two silver electrodes immersed in a liquid. This leads to the production of nanoparticles as a result of their disintegration from the electrode surface without the addition of solvents^10^.

Based on the analysis of the genotoxicity of the silver compounds AgNP, AgNP + C obtained with the HVAD method and AgNO_3_ on the bone marrow cells of chinchilla, carried out using the comet assay, it was demonstrated that each of the tested solutions induced cell DNA damage. There was an increase in the genotoxicity of the solutions in the following order: AgNP < AgNO_3_ < AgNP + C.

The harmful effects of silver nanoparticles in vivo and in vitro studies were investigated using various techniques. Flow cytometry was used^19^, ATP activity measurement^23^ and cytogenetic tests to assess the stability of the genetic material. The analyses of cytotoxicity and genotoxicity were performed using chromosome aberration techniques^13,19^, a micronucleus test^24^ and the comet assay^23,25^.

The comet assay is widely applied in the study of the toxicity of silver nanoparticles. It was used in the analysis of the effects of these particles on both living organisms and cell lines. The advantage of the comet assay is its high sensitivity enabling the identification of damage within a DNA strand, both single-stranded and double-stranded^14^.

Assessing the genotoxic effect of AgNP on cells, the effect of exposure time on changes in the integrity of bone marrow cell DNA was analyzed. A significant relationship was found between the cell exposure time and the increase in DNA helix breakage. A similar relationship was also observed by McShan et al.^22^ in the studies on the effects of AgNP on human keratocytes. He determined the maximum level of DNA damage after 8-h exposure, then followed by a decrease in the DNA integrity disruption after 24 h incubation with AgNP. McShan et al.^22^ suggested that the observed decrease in the percentage of the damaged DNA did not reach the level obtained in the control sample. The research conducted here confirmed the significant impact of exposure time on AgNP activity, showing the highest percentage of DNA in the comet's tail after 6-h exposure. Even a short 3-h exposure of cells to silver compounds resulted in an increase in cell DNA integrity disruption. On the whole, after 24-h exposure of the cells, the lowest degree of genomic instability was determined, which proves that DNA repair mechanisms were activated and that some of the damage was successfully repaired. This confirms the observations of McShan et al.^22^, who found a decrease in the genomic instability of bone marrow cells after 24-h exposure, with the level higher than in control samples. An inverse relationship was observed by Hackenberg et al.^13^, who found significant increase in DNA damage after 24-h exposure as compared to a 3-h one. The lower level of DNA fragmentation after 24 h of exposure indicated the activation of DNA repair mechanisms. However, in the case of exposition to AgNP, complete repair of DNA damage is probably not possible due to the penetration of particles into the cells and the slow release of Ag^+^ ions causing DNA breakage^22^.

Another factor influencing the level of genotoxicity of silver nanoparticles is their concentration in the product. Our research showed a significant relationship between the AgNP concentration and the observed level of DNA integrity disruption. The highest genotoxicity was determined at the concentration of 10 µg/L, a lower one at 20 µg/L, and the least harmful effect was established at the concentration of 5 µg/L. A similar dependency of DNA damage on the dose of AgNP was observed by AshaRani et al.^23^, Hackenberg et al.^13^, Awasthi et al.^12^. In a study by Awasthi et al.^12^, it was observed that the greatest increase in %DNA parameters in the comet tail and the tail moment occurred at the highest doses of AgNP. AshaRani et al.^23^ observed a dose-dependent increase in the value of the tail in relation to the control samples. Ghosh et al.^19^ reported higher %DNA tail values in all concentrations compared to controls. They recorded a rapid increase in the level of DNA helix breaks at a lower concentration of nanoparticles. At the same time, they noted a continuous decrease in DNA instability accompanying an increase in AgNP concentration^19^. The author’s own observations partially confirm these results, showing the highest level of DNA fragmentation at 10 µg/L, while the increase in AgNP and AgNO_3_ concentration to 20 µg/L resulted in a reduction of DNA integrity disruption. The decrease observed may result from the activation of genetic cell defense, in the form of a repair system triggered when a high level of intoxication caused by DNA integration disruption is detected.

In the conducted studies, the genotoxicity of silver nanoparticle compounds was compared with silver nitrate, more precisely with silver ions released by it. In the case of the solutions used, Ag^+^ ions showed a slightly lower toxicity compared to those stabilized with citrate AgNP. Piao et al.^26^ demonstrated higher genotoxicity of silver nanoparticles in relation to silver ions, observing higher values of comet assay parameters for AgNP-treated cells. Conversely, Gliga et al.^20^ and Butler et al.^5^ observed greater toxicity of Ag^+^ ions compared to AgNP. Butler et al.^5^ found that silver ions induced a greater number of DNA helix breaks than silver nanoparticles at the same concentration. However, he observed this relationship especially at lower concentrations. These conclusions can confirm the author's own observations that at lower concentrations, AgNO_3_ caused more disruption of DNA integrity than both the investigated solutions of silver nanoparticles. Greater genotoxicity of Ag^+^ ions derived from AgNO_3_ was also determined in most of the tested exposure times.

Among the research on the genotoxicity of metal nanoparticles, including silver, animals classified as rodents are an experimental model. Both in vitro and in vivo tests are carried out on them. The most commonly used model species in toxicity studies are mice^12,19^ and rats^11,27^. In this study, another rodent species was used as a model, the chinchilla, which, due to its mild disposition and easy breeding, becomes a laboratory animal^28^. These animals are a common model in the study of the nervous and otolaryngological systems^29^. On the other hand, histopathological studies assessing dermal exposure to AgNP were carried out on two species belonging to lagomorphs—rabbits^30^ as well as other species of rodents—domestic *Cavia*^31,32^.

Preliminary genotoxicity analysis of silver nanoparticles produced by the physical method, HVAD, showed a significant effect of these particles on inducing DNA helix breaks. Both the exposure time and AgNP concentration, as well as the interaction of these two factors influence the level of the harmfulness of these solutions. Citrate stabilized AgNP (AgNP + C) showed the greatest effect on the level of induced DNA integrity disruption. Genome instabilities caused by AgNO_3_ did not differ significantly from those caused by AgNP + C. Due to the physical method of obtaining AgNP and the wide variability of the data given in literature on the silver nanoparticles used, their different sizes, coatings and concentrations, the toxicity of the tested solutions cannot be unequivocally compared. Due to their properties, AgNP obtained by the HVAD method can be used as an alternative to chemically synthesized particles.

On the basis of the research conducted, it was determined that there is a need for further tests to compare the toxicity of silver nanoparticles produced by chemical and physical methods, focusing particularly on the concentration, exposure time and the influence of the species.

## Material and methods

### Material: animals

The material for the research was bone marrow isolated from the femurs of 12 chinchilla (*Chinchilla lanigera*) individuals of the Standard strain. The biological material was collected *post mortem*, from the carcasses. The animals were of the same age (1 year). All animals came from the same farm in the south of Poland and were kept in identical standard breeding cages. The chinchillas were kept in accordance with the European Convention for the Protection of Vertebrate Animals, complying with the conditions stipulated in the Act of the 29th of June 2007, presently in force in Poland. Ethics committee approval is not required for the use of biological material collected *post mortem* from animals previously subjected to breeding procedures conducted on livestock farms. At the same time, we emphasize that no animal was sacrificed for the purpose of conducting this experiment.

### Material: silver nanoparticles characteristic

**Figure 5:**
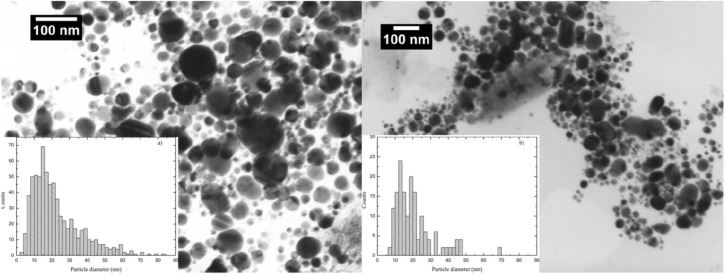
SEM—images and size distribution of silver nanoparticles in water (left) and in TSC (right).

Silver nanoparticles were produced using a high-voltage discharge in an electric arc^10,33^. In the reactor of about 1 × 10^–5^ m^3^ in capacity, two electrodes were placed, 10 mm and 5 mm in diameter, made of 99.9% pure silver, immersed in twice-distilled water (conductivity—(6–10)∙10^−6^ S/cm) or 3.3 µM water solutions of tri-Sodium citrate dihydrate (TSC), (pure, POCH, Gliwice, Poland). The electrical voltage between the electrodes was 20 kV. The distance between the electrodes was 2.5 × 10^−4^ m. Using the dynamic light scattering technique, scanning electron microscope imaging and analyzing of UV–Vis absorption spectrum the size of nanoparticles in water was determined to be about 22 nm and about 38 nm in TSC (Fig. 5). The zeta potential in colloids was estimated at: − 22 mV for water and − 19 mV for TSC.

### Methods: isolation of bone marrow

Bone marrow cells were isolated based on the methodology described by Tjio and Whang^34^. The isolated femurs were cleaned and the epiphyses were cut off. The red bone marrow was rinsed with 2 ml of PBS solution, warmed to 37 °C. Bone marrow cells were exposed to the tested silver compounds, taking into account the variants of concentration and the duration of exposure.

### Methods: cells exposition to silver nanoparticles

The experiment was performed on the control sample (cells not exposed to the tested agents), as well as the cells exposed to the tested silver solutions. The solutions analyzed were as follows: unstable silver nanoparticles in distilled water (AgNP), silver nanoparticles in distilled water stabilized with sodium tricitrate solution (AgNP + C) and silver nitrate as an indicator of toxicity at three concentrations (5,10,20 µg/L) during 3, 6 and 24 h exposure. Silver nanoparticles concentration in colloids was measured using AAS spectrometer.

### Methods: single cell gel electrophoresis assay

Following the exposure, the isolated bone marrow cells were subjected to a comet assay. The alkaline comet assay was performed according to the methodology by Singh et al.^35^. Following the exposure to the tested compounds, the unfixed bone marrow cells were suspended in 1% low melting point (LMP) agarose (Sigma) and placed on slides coated with 1% Normal Melting Point (NMP) Agarose (Sigma). The preparations were lysed for 24 h in the buffer of 2.5 M NaCl (Sigma), 0.1 M EDTANa2 (Sigma), 10 mM TRIS (Sigma), 1% Triton X-100 pH = 10 (Sigma) at 4^o^ C. The preparations were then incubated for 20 min in a chilled electrophoresis buffer containing 30 mM NaOH (Sigma), 2 mM EDTANa2, pH = 12.5. The electrophoresis was performed for 20 min at 0.6 V/cm. For the purpose of the analysis, the slides were stained with ethidium bromide, at a concentration of 200 µg/ml. To determine DNA damage, the percentage of DNA in the comet tail (% of DNA in the tail, TD%) and the tail moment (TM) were analyzed. It is a composite parameter, calculated as the product of the percentage of DNA content in the comet tail and the length of the comet tail.

### Microscopic analysis

Microscopic analysis and photographic documentation were prepared using a Zeiss Imager A2 epifluorescence microscope coupled with a Zeiss AxioCam MRc5 digital camera and NIS-Elements image analysis software ver. F2.31.

### Statistical analysis

In the experiment, the following factors were tested: the type of solution, the concentration of the solution, and the exposure time. The influence of these factors on the percentage of DNA in the tail and the tail moment (TM) was analyzed. For this purpose, mean values of measurements made in 12 individuals were used. At the first stage of the analysis, the influence of the factors examined was compared with the control sample using the Student's t-test. At the second stage of statistical analysis, a three-factor analysis of variance (ANOVA) was used to assess the effects of each solution type (factor A), solution concentration (factor B), as well as exposure time (factor C) on the characteristics tested. The analysis was conducted in accordance with the following mathematical model:

$${\text{y}}_{{{\text{ijlk}}}} = {\text{m}} + {\text{a}}_{{\text{i}}} + {\text{b}}_{{\text{j}}} + {\text{c}}_{{\text{l}}} + {\text{ab}}_{{{\text{ij}}}} + {\text{ac}}_{{{\text{il}}}} + {\text{bc}}_{{{\text{jl}}}} + {\text{abc}}_{{{\text{ijl}}}} + {\text{e}}_{{{\text{ijlk}}}}$$

where y_ijlk_—value of the tested feature, m—population mean, a_i_—influence of the i-th level of factor A (type of solution), b_j_—effect of the j-th level of factor B—(solution concentration), c_l_—the effect of the l-th level of factor C (exposure time), ab_ij_—effect of the interaction of factors A and B (type of solution and concentration of solution), ac_il_—effect of the interaction of factors A and C (type of solution and exposure time), bc_jl_—effect of the interaction of factors B and C (solution concentration and exposure time), abc_ijl_—effect of the interaction of factors A, B, C (type of solution, concentration of solution and exposure time), e_ijlk_—random error.

Tukey’s range test was used to compare the means. All calculations were performed at *p* ≤ 0.05.

## Abbreviations

AgNP: Silver nanoparticles
HVAD: High voltage arc discharge
SCGE: Single cell gel electrophoresis
SSB: Single strand break
DSB: Double strand break
AgNP + C: Silver nanoparticles with sodium tricitrate
TD%: % of DNA in the comet’s tail
TM: Tail moment
